# Enhancing Performance of a MEMS-Based Piezoresistive Pressure Sensor by Groove: Investigation of Groove Design Using Finite Element Method

**DOI:** 10.3390/mi13122247

**Published:** 2022-12-17

**Authors:** Phongsakorn Thawornsathit, Ekachai Juntasaro, Hwanjit Rattanasonti, Putapon Pengpad, Karoon Saejok, Chana Leepattarapongpan, Ekalak Chaowicharat, Wutthinan Jeamsaksiri

**Affiliations:** 1 Mechanical Engineering Simulation and Design Group, The Sirindhorn International Thai-German Graduate School of Engineering (TGGS), King Mongkut’s University of Technology North Bangkok, Bangsue, Bangkok 10800, Thailand; 2 Thai Microelectronics Center (TMEC), National Electronics and Computer Technology Center, National Science and Technology Development Agency, 24000 Chachoengsao, Thailand

**Keywords:** MEMS piezoresistive pressure sensor, groove, sensitivity, linearity, FEM

## Abstract

The optimal groove design of a MEMS piezoresistive pressure sensor for ultra-low pressure measurement is proposed in this work. Two designs of the local groove and one design of the annular groove are investigated. The sensitivity and linearity of the sensor are investigated due to the variations of two dimensionless geometric parameters of these grooves. The finite element method is used to determine the stress and deflection of the diaphragm in order to find the sensor performances. The sensor performances can be enhanced by creating the annular or local groove on the diaphragm with the optimal dimensionless groove depth and length. In contrast, the performances are diminished when the local groove is created on the beam at the piezoresistor. The sensitivity can be increased by increasing the dimensionless groove length and depth. However, to maintain low nonlinearity error, the annular and local grooves should be created on the top of the diaphragm. With the optimal designs of annular and local grooves, the net volume of the annular groove is four times greater than that of the local groove. Finally, the functional forms of the stress and deflection of the diaphragm are constructed for both annular and local groove cases.

## 1. Introduction

Microelectromechanical system (MEMS) pressure sensors occupy the largest market share in the world market of MEMS devices [1,2,3]. They have been widely used in various fields, such as the automotive industry [4,5], the aerospace industry [6,7], biomedical applications [8,9] and the household appliances [10,11]. From the market trends, the report of MEMS pressure sensors shows that from 2019 to 2026, the demands of MEMS pressure sensors in biomedical applications (invasive measurements) and the industrial market (factory automation, process control systems and smart meters) will be expanding by around 30% and 50%, respectively, which are the top two expansion rates of demand in the MEMS pressure sensor market, according to Damianos and Mouly (2021) [12]. The ultra-low pressure measurement is required in both biomedical applications (2–4 kPa) [13] and process control systems such as HVAC (heating, ventilation and air conditioning) controls (0.1–1 kPa) [11]. There are two main types of MEMS pressure sensors that are widely used for the ultra-low pressure measurement, i.e., the piezoresistive pressure sensor and the capacitive one. To measure ultra-low pressure, high sensitivity, high linearity and stability are required in the MEMS pressure sensors [2,11]. The MEMS capacitive pressure sensor has the advantages of the simplicity of the technological route, high sensitivity, low energy consumption and low temperature sensitivity, but low linearity, low stability and high vibration sensitivity are its main disadvantages [14,15]. Moreover, the external processing circuit or application-specific integrated circuit (ASIC) is also required for converting the capacitance to voltage. Although the MEMS capacitive pressure sensor for low pressure range was able to give a high sensitivity of about 260 aF/Pa with a fine resolution of about 0.025% in full-scale range, its nonlinearity error was found to be quite high, i.e., around −2% FSS [15]. Therefore, for ultra-low pressure measurements, when high accuracy is required, the MEMS piezoresistive pressure sensor is preferred. The MEMS piezoresistive pressure sensor has the advantages of high linearity and stability [2], while its sensitivity can be improved by changing the diaphragm geometry, as reported in several previous works. Zhao et al. (2016) [16] proposed a bossed diaphragm with a peninsula-island structure, which was able to give sensitivity of 0.066 mV/V/kPa and nonlinearity error of 0.42% FSS. Guan et al. (2016) [17] obtained sensitivity of 4.72 mV/V/kPa and nonlinearity error of 0.18% FSS by designing the shuriken-structure diaphragm. Xu et al. (2017) [18] proposed the diaphragm with groove and peninsula island, which provided sensitivity of 60 mV/V/kPa and nonlinearity error of 0.36% FSS. Tran et al. (2018a) [19] proposed a combination of the cross-beam membrane and the peninsula (CBMP), which gave sensitivity of 5.4 mV/V/kPa and nonlinearity error of 0.28% FSS. Tran et al. (2018b) [20] proposed the diaphragm with a combination of the petal edge, the narrow beam, the center boss and the groove, which provided sensitivity of 6.93 mV/V/kPa and nonlinearity error of 0.23% FSS. Li et al. (2020) [21] proposed the diaphragm with groove and rood beam, which provided sensitivity of 4.48 mV/V/kPa and nonlinearity error of 0.25% FSS. Zoheir et al. (2020) [22] proposed the diaphragm with a patterned groove, which was able to provide sensitivity of 2.1 mV/V/kPa. Basov and Prigodskiy (2020) [23] proposed the diaphragm with multirigid islands, which gave sensitivity of 34.5 mV/V/kPa and nonlinearity error of 0.81% FSS. Basov (2021) [24] proposed the novel electrical circuit, which was able to provide sensitivity of 44.9 mV/V/kPa and nonlinearity error of 1.2% FSS. The main challenge of the diaphragm design of the MEMS piezoresistive pressure sensor is to achieve a tradeoff between sensitivity and linearity. There are several techniques for the diaphragm design to improve sensitivity with less of a negative effect on linearity, as will be described in Section 2.2 (design considerations). The groove design is one of the interesting techniques for the diaphragm design that is investigated in the present work in order to find the optimal groove design(s) for the MEMS piezoresistive pressure sensor for ultra-low pressure measurements.

## 2. Background

### 2.1. Working Principle of MEMS Piezoresistive Pressure Sensors

The MEMS piezoresistive pressure sensor can detect pressure by realizing the effect of piezoresistance on the piezoresistors, where the relative change in resistance $\left(\Delta R/R_{0}\right)$ can be expressed as [25]

(1) $$\frac{\Delta R}{R_{0}}=\sigma_{l}\pi_{l}+\sigma_{t}\pi_{t}$$

where $\sigma_{l}$ and $\sigma_{t}$ are the longitudinal and transverse stresses within the piezoresistor, respectively, and $\pi_{l}$ and $\pi_{t}$ are the longitudinal and transverse piezoresistance coefficients, respectively. In the present work, four p-type piezoresistors are fabricated on the (100) oriented plane along the <110> direction. Therefore, $\Delta R/R_{0}$ in Equation (1) can be expressed, according to [26], as

(2) $$\frac{\Delta R}{R_{0}}=\frac{\pi_{44}\left(\sigma_{l}-\sigma_{t}\right)}{2}=\frac{\pi_{44}\left(\Delta \sigma \right)}{2}$$

where Δσ is the stress difference between the longitudinal and transverse stresses within the piezoresistor and $\pi_{44}=138.1\times 10^{-11}\;{\mathrm{Pa}}^{-1}$, according to Bao (2005) [27]. In the present work, four silicon piezoresistors are arranged in the full Wheatstone bridge circuit, and the output voltage $\left(V_{out}\right)$ can be described as

(3) $$V_{out}=\left[\frac{\Delta \sigma_{1}-\Delta \sigma_{2}}{\left(4/\pi_{44}\right)+\Delta \sigma_{1}+\Delta \sigma_{2}}\right]V_{in}$$

where $V_{in}$ is the supply voltage and subscripts 1 and 2 denote the variables of the resistor $R_{1}$ and $R_{2}$, respectively.

### 2.2. Design Considerations

The MEMS piezoresistive pressure sensor for ultra-low pressure measurement in applications, such as biomedical devices and HVAC systems, requires high sensitivity (S) and low nonlinearity error (NL). The sensitivity is defined as the ratio of the full-span scale output voltage ($V_{FSS}$) to the pressure difference (ΔP) between the maximum applied pressure ($P_{max}$) and the reference pressure ($P_{0}$) divided by $V_{in}$, which can be expressed as

(4) $$S=\frac{V_{max}-V_{offset}}{\left(P_{max}-P_{0}\right)\cdot V_{in}}=\frac{V_{FSS}}{\Delta P\cdot V_{in}}$$

The nonlinearity error represents the accuracy of the sensor, whose definition is the percentage of the output voltage difference (ΔV) between the output voltage at the measurement point ($V_{out,i}$) and the ideal output voltage ($V_{ideal}$) normalized by $V_{FSS}$. The nonlinearity error can be written as

(5) $$NL=100\%\times \frac{\left[V_{out,i}-V_{ideal}\right]}{V_{FSS}}=100\%\times \frac{\Delta V}{V_{FSS}}$$

where $V_{ideal}=V_{offset}+\left(P_{i}-P_{0}\right)\left(\frac{V_{FSS}}{\Delta P}\right)$. The highest nonlinearity error of all measurement points is used to represent the nonlinearity error of the sensor in this work.

On the basis of the basic design of the flat diaphragm, sensitivity is higher when the width to thickness ratio (b/j) of the square diaphragm is increased but nonlinearity error is also increased as a consequence, where b is the diaphragm width and j is the diaphragm thickness. There are two main sources of nonlinearity error. The first source is the nonlinearity of stress when the balloon effect occurs [8]; it is well known that the stiffness at the diaphragm edge is higher than that in the middle of the diaphragm. Therefore, when the thin diaphragm is exerted by high pressure, the balloon effect can occur. The second source is the unbalanced stresses between $R_{1}$ and $R_{2}$ ($\left|\Delta \sigma_{1}\right|\neq \left|\Delta \sigma_{2}\right|$). The difference in the mechanical stresses of the resistors can be caused by the inappropriate placement of the piezoresistors or technological errors, such as the method of diaphragm etching, the error in lithography in displacement of Wheatstone bridge branches, the quality of the orientation of the crystallographic plane and the direction in the original wafers as a material, which leads to uneven etching, asymmetry in the stepped arrangement of the dielectric layers on chip surface, etc. According to Equation (3), if $\Delta \sigma_{1}+\Delta \sigma_{2}$ is nonzero, $V_{out}$ is not linearly proportional to the stress even though $\Delta \sigma_{1}$ and $\Delta \sigma_{2}$ change linearly with the pressure [28]. To alleviate the balloon effect, the local stiffness of the diaphragm is taken into account when the diaphragm is designed. The flexural stiffness (D) of the diaphragm is a function of the diaphragm thickness (j), which is described as [29]

(6) $$D=\frac{Ej^{3}}{12\left(1-\nu^{2}\right)}$$

where E is Young’s modulus and ν is Poisson’s ratio. Therefore, the diaphragm thickness is a key geometric parameter for the diaphragm design. Sandmaier (1991) [30] increased the local thickness in the middle of the diaphragm by additionally attaching the boss. The boss can help reduce deflection (δ), and as a result, the balloon effect and the nonlinearity error are reduced. To obtain higher sensitivity, the diaphragm width (b) must be made larger. Therefore, the diaphragm width is one of the key factors in design of MEMS piezoresistive pressure sensors, especially in biomedical devices. Instead of having the boss in the middle of the diaphragm, using the cross-beam on the diaphragm was proposed by Tian et al. (2010) [31]. For the diaphragm with the cross-beam, the diaphragm width becomes smaller without loss of sensitivity and linearity, compared with the diaphragm with boss. With the same diaphragm width as in Tian et al. (2010) [31], Huang and Zhang (2014) [28] proposed the peninsula structures that are located at four sides of the diaphragm edge. The diaphragm with the peninsula structures can achieve twice the sensitivity with slightly increased nonlinearity error, compared with the diaphragm with the cross-beam. To obtain higher sensitivity, the stress within SCR, where the piezoresistors are placed, must be increased. SCR is commonly created by an abrupt change in the geometry’s cross-sectional area, typically around a sharp corner, hole, notch or groove. When the size of SCR is reduced, the strain energy is generated within the smaller volume of SCR, and hence, higher strain-energy density occurs in SCR, leading to the increased stress [32]. Many research works reported that higher sensitivity could be achieved by creating a groove. Shimazoe and Matsuoka (1982) [33] reported that a combination of the circular diaphragm with center boss and annular groove significantly improved both sensitivity and linearity compared with the conventional diaphragm. However, the chip size was large and the demand on higher sensitivity was still required. Zhang et al. (2014) [34] reported that the annular groove diaphragm with embedded silicon nanowires could boost sensitivity by 1.78 times compared with their previous work [35], but the effect of the annular groove on sensitivity and linearity was not presented. Xu et al. (2016) [36] proposed the high-sensitivity pressure sensor for the pressure range of 0–500 Pa. To obtain higher sensitivity, the annular groove depth was optimized by factoring in the averaged stress difference and the frequency of the first resonance mode, but the effect of the groove depth on nonlinearity error was not presented. Li et al. (2017) [37] proposed the annular groove diaphragm with the rood beam, which could improve sensitivity, but the effect of the groove dimensions on sensitivity and linearity was not clearly reported. Sahay et al. (2021) [38] proposed a combination of the annular groove diaphragm with center boss, which helped improve both sensitivity and linearity, but the information on how to obtain the proposed groove dimension was not presented. Zoheir and Sajjad (2018) [39] investigated the groove design for the MEMS cantilever-based energy harvester, in which the position and configuration of groove were the main factors that significantly affected the output voltage. Tran et al. (2018b) [20] increased the stress in SCR by changing the diaphragm edge to the petal shape and also added the local groove in order to increase sensitivity. It was found that the local groove at the longitudinal piezoresistor was the best configuration to acquire the best sensor performance, but the effect of the groove dimension on sensitivity and linearity was not presented. Zoheir et al. (2020) [22] proposed a diaphragm with a patterned groove, which could significantly improve sensitivity, but the nonlinearity error of the sensor was not presented. According to previous works [20,22,33,34,36,37,38,39], it was found that the diaphragm with groove was able to achieve higher sensitivity. However, how the groove is to be properly designed was not yet clear in previous works. Therefore, three groove designs are investigated in the present work.

### 2.3. Groove Designs

**Figure 1:**
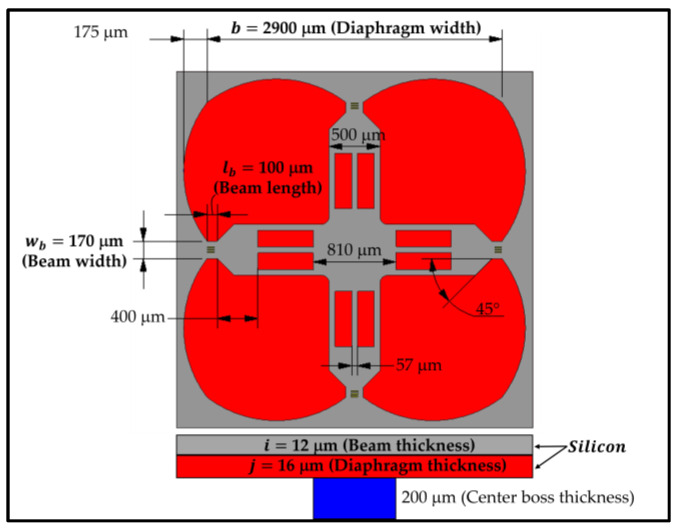
MEMS piezoresistive pressure sensor for ultra-low pressure measurements with optimized geometric parameters, proposed by Thawornsathit et al. (2022) [40].

Because the MEMS piezoresistive pressure sensor for ultra-low pressure measurements with optimized geometric parameters of boss, cross-beam, peninsular structures and petal edge was proposed by Thawornsathit et al. (2022) [40], as shown in Figure 1, each groove design in the present work is investigated on the framework of the MEMS piezoresistive pressure sensor of Thawornsathit et al. (2022) [40].

To design the groove, there are three important geometric parameters, i.e., the groove width $\left(w_{g}\right)$, the groove depth $\left(d_{g}\right)$ and the groove length $\left(l_{g}\right)$, that should be considered for how they affect the stress (σ) in the piezoresistor and the maximum deflection of the diaphragm $\left(\delta_{max}\right)$. In order to determine the significant geometric parameters for the analysis and construction of the scaling law, the geometric parameters are expressed in dimensionless forms. The reasoning for constructing each dimensionless geometric parameter is explained as follows. The dimensionless groove width is expressed as $\bar{w_{g}}=w_{g}/l_{b}$, where $l_{b}$ is the beam length. Because the purpose of making the groove is to increase σ in SCR with effect on $\delta_{max}$ as less as possible, the groove area should be kept as small as possible. SCR is found on the beam where the piezoresistor is placed so that $w_{g}$ should be bounded by $l_{b}$. In the present work, all groove designs are investigated in case of $\bar{w_{g}}$ = 1 when $l_{b}$ = 100 μm, as shown in Figure 1. To construct the dimensionless groove depth $(\bar{d_{g}}$), $d_{g}$ is nondimensionalized by the thickness of the layer upon which the groove is created so that $\bar{d_{g}}$ is varied between 0 and 1 for all case studies. The dimensionless groove length $(\bar{l_{g}}=l_{g}/w_{b}$) is constructed by nondimensionalizing $l_{g}$ with the beam width $\left(w_{b}\right)$—in other words, the ratio of the groove length to the length of SCR. σ and $\delta_{max}$ are also considered in dimensionless forms of $\bar{\sigma }$ and $\bar{\delta }$, respectively, which are explained as follows. According to the Kirchoff–Love plate theory [41], the governing equation of the diaphragm deflections can be written in the Cartesian coordinates as

(7) $$\left(\frac{\partial^{4}\delta }{\partial x^{4}}+2\frac{\partial^{4}\delta }{\partial x^{2}\partial y^{2}}+\frac{\partial^{4}\delta }{\partial y^{4}}\right)=\frac{P}{D}$$

where δ is governed by D and P. Intuitively, δ is also dependent on b because in principle b is used to nondimensionalize the coordinates (x, y). According to this observation and the aid of the definition of D in Equation (6), Clark and Wise (1979) [29] proposed the dimensionless diaphragm deflection as

(8) $$\bar{\delta }=\frac{Ej^{3}\delta }{12\left(1-\nu^{2}\right)Pb^{4}}$$

This dimensionless diaphragm deflection is adopted in this work. The stress components $\sigma_{x}$ and $\sigma_{y}$ in the x and y directions, respectively, and the shear stress $\tau_{xy}$ on the diaphragm can be defined as

(9) $$\sigma_{x}=-\frac{Ej}{2\left(1-\nu^{2}\right)}\left(\frac{\partial^{2}\delta }{\partial x^{2}}+\nu \frac{\partial^{2}\delta }{\partial y^{2}}\right)$$

(10) $$\sigma_{y}=-\frac{Ej}{2\left(1-\nu^{2}\right)}\left(\nu \frac{\partial^{2}\delta }{\partial x^{2}}+\frac{\partial^{2}\delta }{\partial y^{2}}\right)$$

(11) $$\tau_{xy}=Gj\left(\frac{\partial^{2}\delta }{\partial x\partial y}\right)$$

where G is the shear modulus. These stresses are dependent on δ. Hence, these stresses are also dependent on D, P and b. When the buckling diaphragm is considered, the stresses depend on the square of the ratio of the diaphragm thickness to its width $\left(j^{2}/b^{2}\right)$, according to Clark and Wise (1979) [29]. Therefore, $\bar{\sigma }$ can be nondimensionalized as

(12) $$\bar{\sigma }=\frac{\sigma j^{2}}{Pb^{2}}$$

Because $\bar{w_{g}}$ is fixed as a constant in all case studies in the present work, the behaviors of $\bar{\delta }$ and $\bar{\sigma }$ are investigated as the functions of only two dimensionless parameters, i.e., $\bar{d_{g}}$ and $\bar{l_{g}}$. Moreover, the effects of $\bar{d_{g}}$ and $\bar{l_{g}}$ on the sensitivity and nonlinearity of the MEMS piezoresistive pressure sensor are also investigated in each groove design.

**Figure 2:**
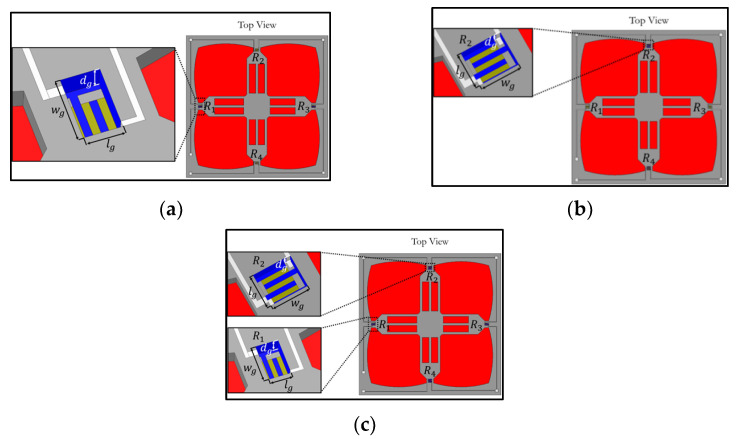
Local groove design 1: (**a**) grooves at longitudinal piezoresistors (LG1−L0), (**b**) grooves at transverse piezoresistors (LG1−0T) and (**c**) grooves at locations of both longitudinal and transverse piezoresistors (LG1−LT ). (Note: not to scale.)

According to several previous works [20,22,33,34,36,37,38,39], two groove types were found in the MEMS piezoresistive pressure sensor: the local groove and the annular groove. Therefore, two designs of the local groove and one design of the annular groove are investigated in this work. The first design is the local groove LG1, proposed by Tran et al. (2018b) [20], where grooves are created at the locations of the piezoresistors, as shown in Figure 2.

**Figure 3:**
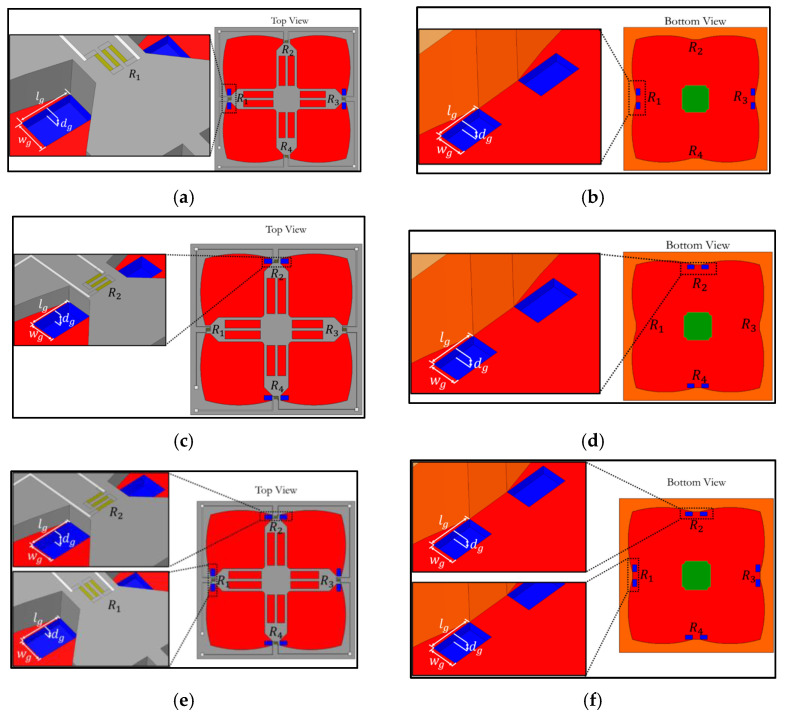
Local groove design 2: (**a**) grooves on top of diaphragm at longitudinal piezoresistors (LG2−L0−Top), (**b**) grooves at bottom of diaphragm at longitudinal piezoresistors (LG2−L0−Bottom), (**c**) grooves on top of diaphragm at transverse piezoresistors (LG2−0T−Top), (**d**) grooves at bottom of diaphragm at transverse piezoresistors (LG2−0T−Bottom), (**e**) grooves on top of diaphragm at both longitudinal and transverse piezoresistors (LG2−LT−Top ) and (**f**) grooves at bottom of diaphragm at both longitudinal and transverse piezoresistors (LG2−LT−bottom ). (Note: not to scale.)

The second design is also the local groove LG2, proposed in this work, where grooves are created on the diaphragm along both sides of the beams, as shown in Figure 3. To find the proper design of the local groove LG2 that is comparable to the local groove LG1 on the same basic, two criteria for the dimensions of local groove LG2 are specified in the present work, as follows: (1) $\bar{l_{g}}$ = 0.175, which is specified when the dimensionless net groove volume of LG2, i.e., for eight grooves $\left(8\times \bar{l_{g}}\times \bar{d_{g}}\times \bar{w_{g}}\right)$, is equal to that of LG1, i.e., for four grooves $\left(4\times \bar{l_{g}}\times \bar{d_{g}}\times \bar{w_{g}}\right)$, and (2) $\bar{l_{g}}$ = 0.35, which is specified when the dimensionless groove volume of LG2 $\left(\bar{l_{g}}\times \bar{d_{g}}\times \bar{w_{g}}\right)$ is equal to that of LG1 $\left(\bar{l_{g}}\times \bar{d_{g}}\times \bar{w_{g}}\right)$. 

**Figure 4:**
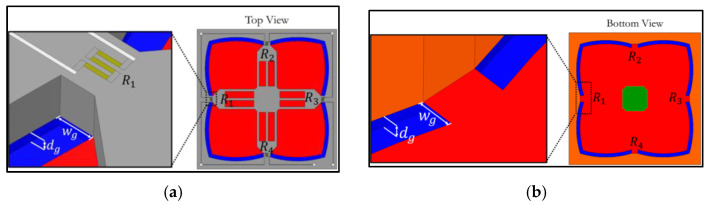
Annular groove: (**a**) grooves on top of diaphragm (AG−Top) and (**b**) grooves at bottom of diaphragm (AG−Bottom). (Note: not to scale.)

The third design is the annular groove AG, where grooves are created along the diaphragm edge, as shown in Figure 4. For LG1 and LG2, grooves are created at three locations, as follows: (1) grooves only at longitudinal piezoresistors (LGX−L0), (2) grooves only at transverse piezoresistor locations (LGX−0T) and (3) grooves created at both longitudinal and transverse piezoresistors (LGX−LT). The comparison of sensor performances of grooves on the top of the diaphragm with grooves at the bottom of the diaphragm are also investigated in this work, as shown in Figure 3 and Figure 4. Therefore, there are 11 groove configurations investigated in this work, as listed in Table 1.

## 3. Finite Element Analysis

**Figure 5:**
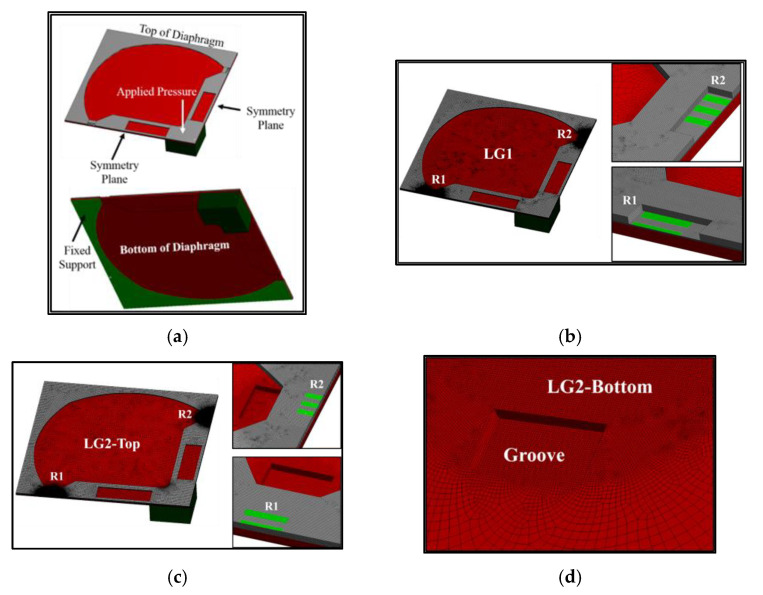

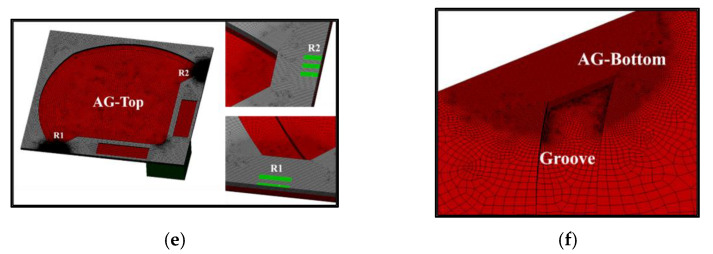
Computational domain for finite element analysis: (**a**) boundary conditions, (**b**) mesh distribution for LG1, (**c**) mesh distribution for LG2-Top, (**d**) mesh distribution for LG2-Bottom, (**e**) mesh distribution for AG-Top and (**f**) mesh distribution for AG-Bottom.

To determine the stress and deflection of the diaphragm over a range of the applied pressures of 1–5 kPa, the finite element method is performed by using the commercial software ANSYS Mechanical version 18.1. Because of the symmetry in the middle of the sensor, only a quarter of the sensor is created for the finite element model, as shown in Figure 5a. The hexahedral cell type with a quadratic element order is used for all case studies, as shown in Figure 5b–f. The grids are set with the same type in all case studies where there are differences only in the thickness of the grid when the groove depth is changed. Because of the nonlinear mechanical behavior, the large deflection model is used to acquire an accurate result, following Thawornsathit et al. (2022) [40]. For the material properties of silicon used in the simulation for both diaphragm and beam layers, Young’s modulus and Poisson’s ratio are 160 GPa and 0.22, respectively [42,43].

### 3.1. Stress Distribution of the Sensor with the Local Groove LG1

**Figure 6:**
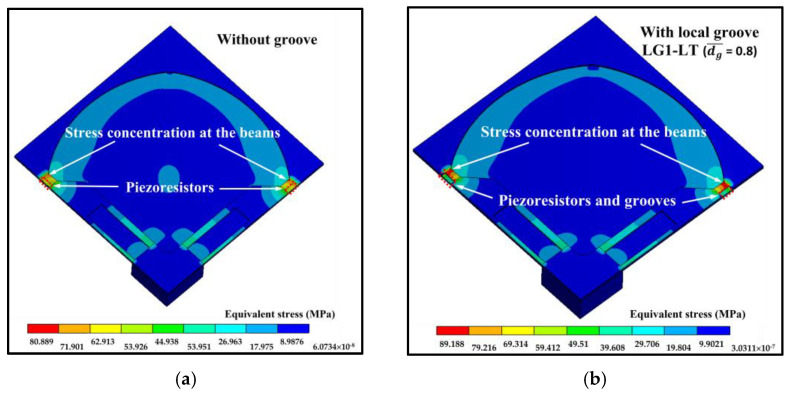
Equivalent stress distributions at the applied pressure of 5 kPa: (**a**) sensor without groove and (**b**) sensor with local groove LG1−LT ($\bar{d_{g}}$ = 0.8). (Only one-fourth of the domain is displayed.)

**Figure 7:**
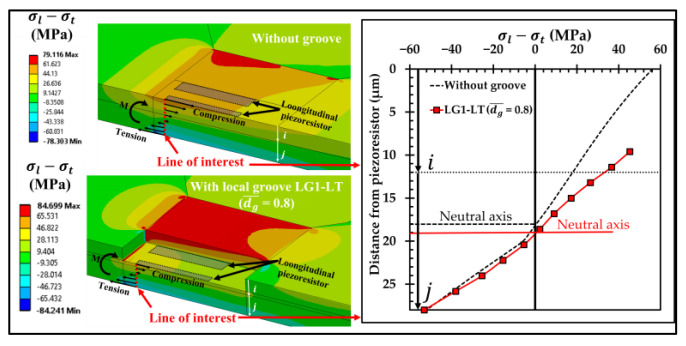
Stress difference distributions at the longitudinal piezoresistor without groove and with local groove LG1−LT ($\bar{d_{g}}$ = 0.8) at the applied pressure of 5 kPa. (Only one-half of the domain is displayed.)

**Figure 8:**
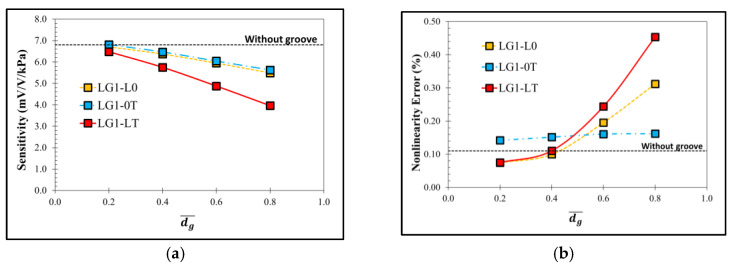
Variations of (**a**) sensitivity and (**b**) nonlinearity error with dimensionless groove depth of sensor with local grove LG1.

**Figure 9:**
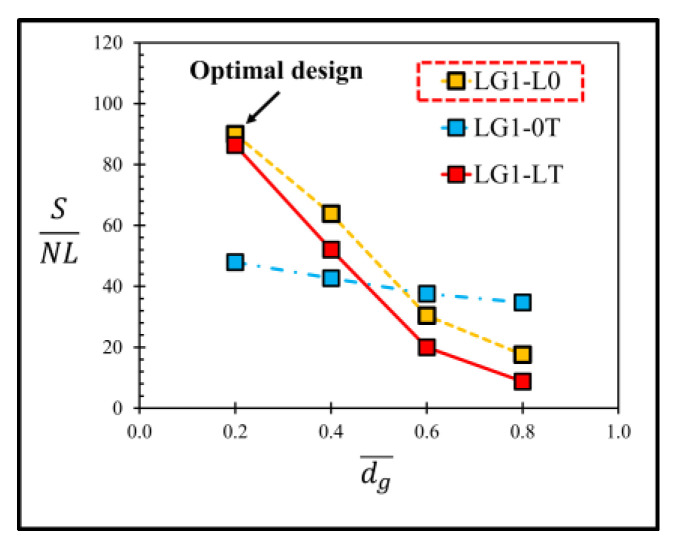
Variation of ratio of sensitivity to nonlinearity error $\left(\frac{S}{NL}\right)$ with dimensionless groove depth of sensor with local grove LG1.

Figure 6 shows the comparison of the equivalent stress distributions at the applied pressure of 5 kPa between the sensor without groove and the sensor with the local groove LG1−LT. The equivalent stress distributions reveal that the stresses at the piezoresistors decrease when the local groove LG1−LT is created. The distributions of the stress difference $\left(\sigma_{l}-\sigma_{t}\right)$ at the piezoresistors with and without groove are studied in detail in Figure 7. Because of the bending moment (M), the compression stress $\left(\sigma_{c}\right)$ is generated above the neutral axis, whereas the tension stress $\left(\sigma_{t}\right)$ is generated below the neutral axis. Figure 7 reveals that the stress difference is close to zero near the neutral axis. Therefore, the stress difference at the piezoresistor placed in the local groove LG1−LT decreases because the piezoresistor is located closer to the neutral axis so that the sensitivity of the sensor with the local groove LG1−LT is decreased, as can be seen in Figure 8a. Figure 8b shows that the nonlinearity error of the sensor with the local groove LG1 drastically increases when $\bar{d_{g}}$ is increased in the case when the local groove LG1 is created at the longitudinal piezoresistors, i.e., LG1−L0 and LG1−LT, whereas the effect of $\bar{d_{g}}$ on the nonlinearity error is negligible when the local groove LG1 is created at the transverse piezoresistors, i.e., LG1−0T. Because sensitivity and nonlinearity error are required to be maximized and minimized, respectively, the maximum ratio of sensitivity to nonlinearity error ${\left(\frac{S}{NL}\right)}_{max}$ is used as a criterion to determine the optimal design of the sensor with groove. Figure 9 reveals that LG1−L0 at $\bar{d_{g}}$ = 0.2 is the optimal design of the local groove LG1.

### 3.2. Stress Distribution of the Sensor with the Local Groove LG2

**Figure 10:**
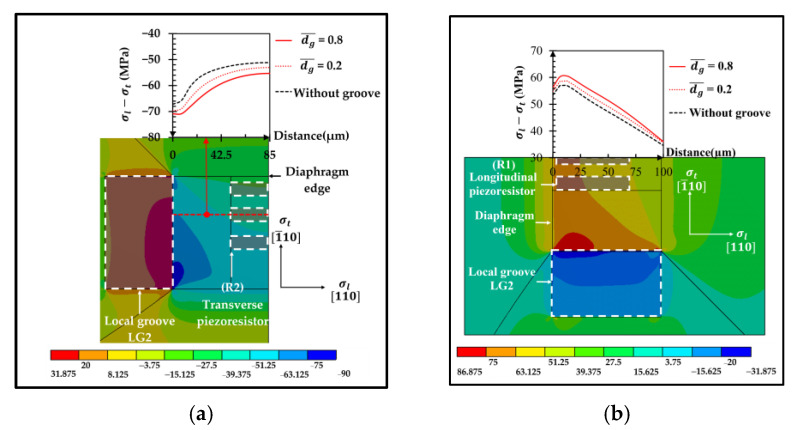
Stress difference distributions at the applied pressure of 5 kPa: (**a**) transverse piezoresistor and (**b**) longitudinal piezoresistor for LG2−LT−Top ($\bar{d_{g}}$ = 0.8). (Only one-half of the domain is displayed.)

**Figure 11:**
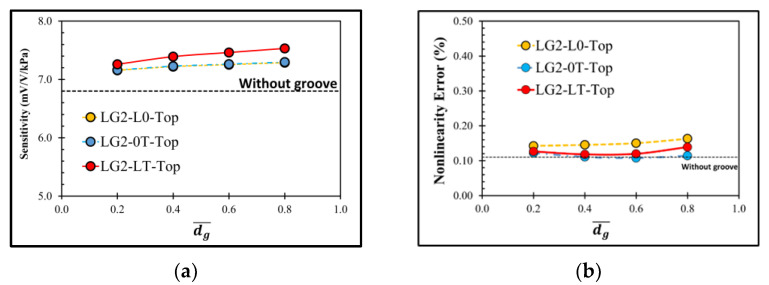
Variations of (**a**) sensitivity and (**b**) nonlinearity error with dimensionless groove depth of sensor with local grove LG2 on the top of diaphragm at $\bar{l_{g}}=0.175$.

Figure 10 shows the distributions of $\sigma_{l}-\sigma_{t}$ at the transverse and longitudinal piezoresistors of the sensor with the local groove LG2−LT on the top of the diaphragm. The increment of $\bar{d_{g}}$ slightly affects the magnitude of $\sigma_{l}-\sigma_{t}$, leading to the small change in sensitivity and nonlinearity error, as shown in Figure 11. When the local groove LG2 is created at only the longitudinal piezoresistors (LG2−L0−Top), the worst nonlinearity error is found.

**Figure 12:**
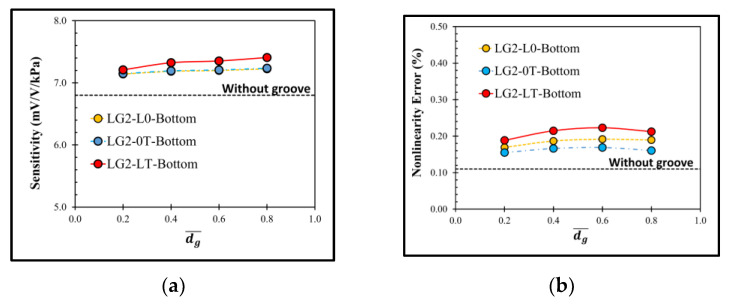
Variations of (**a**) sensitivity and (**b**) nonlinearity error with dimensionless groove depth of sensor with local grove LG2 at the bottom of diaphragm at $\bar{l_{g}}=0.175$.

The performances of the sensor with the local groove LG2 at the bottom of diaphragm is also investigated, as shown in Figure 12. The sensor with the local groove LG2 at the bottom of diaphragm experiences the same effect of $\bar{d_{g}}$ on sensitivity as the sensor with the local groove LG2 on the top of diaphragm; however, its nonlinearity error is worse in all case studies. Therefore, the effect of $\bar{l_{g}}$ on the sensor performances is worth investigating in the case of the sensor with the local groove LG2 on the top of diaphragm only.

**Figure 13:**
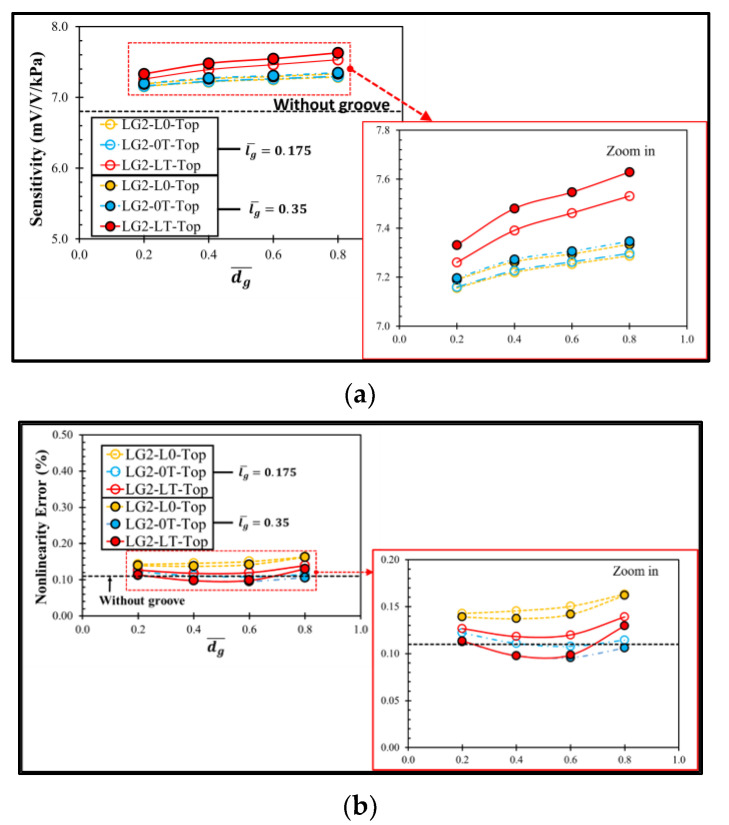
Effects of $\bar{l_{g}}$ on (**a**) sensitivity and (**b**) nonlinearity error of sensor with local groove LG2 on the top of diaphragm.

Figure 13 shows the effect of $\bar{l_{g}}$ on the performances of the sensor with the local groove LG2 on the top of the diaphragm. With the double increment of $\bar{l_{g}}$, the sensitivity can be increased by 0.6% for the local groove LG2 at the longitudinal or transverse piezoresistors, i.e., LG2−L0−Top or LG2−0T−Top, respectively, and 1% for the local groove LG2 at both the longitudinal and transverse piezoresistors, i.e., LG2−LT−Top, and the nonlinearity error is lower in all case studies, especially when the local groove LG2 is created with $0.4\leq \bar{d_{g}}\leq 0.6$. 

**Figure 14:**
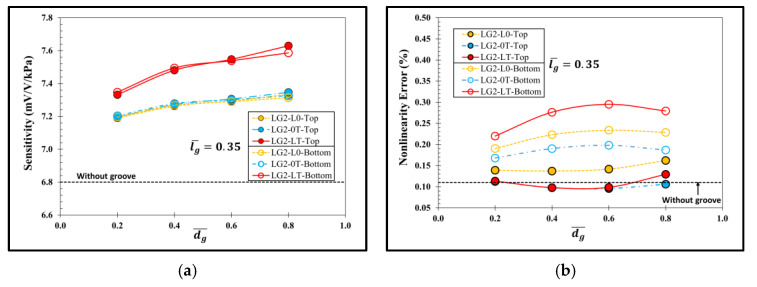
Comparisons of (**a**) sensitivity and (**b**) nonlinearity error between sensors with local groove LG2 and $\bar{l_{g}}$ = 0.35 on top and at bottom of diaphragm.

Figure 14 shows the performance comparisons between the sensor with the local groove LG2 and $\bar{l_{g}}$ = 0.35 on the top and the same at the bottom of the diaphragm. The sensitivity in case of LG2 on the top of diaphragm is higher than that of LG2 at the bottom of diaphragm when $\bar{d_{g}}>0.4$, whereas the linearity in the case of LG2 on the top of diaphragm is better than that of LG2 at the bottom of diaphragm in all case studies.

**Figure 15:**
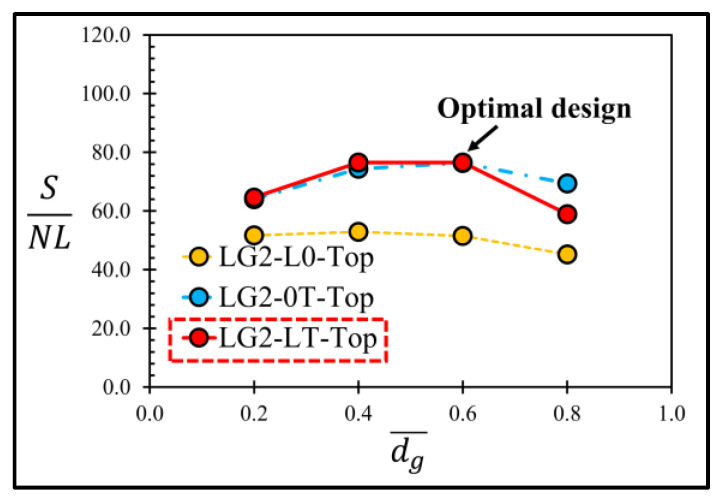
Variation of ratio of sensitivity to nonlinearity error $\left(\frac{S}{NL}\right)$ with dimensionless groove depth of sensor with local grove LG2 at $\bar{l_{g}}=0.35$.

Therefore, the sensor with the local groove LG2 on the top of diaphragm and $\bar{l_{g}}$ = 0.35 is investigated further to determine its optimal design by considering ${\left(\frac{S}{NL}\right)}_{max}$. Figure 15 reveals that LG2−LT−Top at $\bar{d_{g}}$ = 0.6 is the optimal design of the local groove LG2.

### 3.3. Stress Distribution of the Sensor with the Annular Groove AG

**Figure 16:**
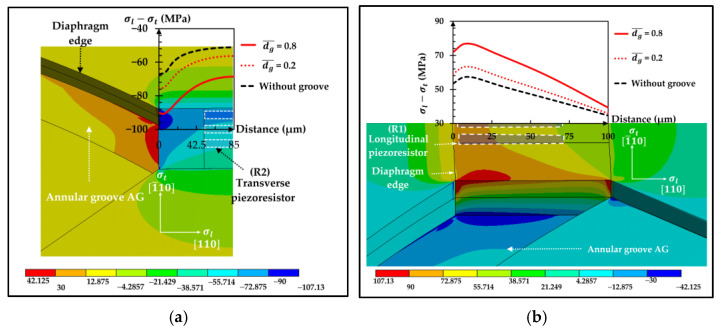
Stress difference distributions at the applied pressure of 5 kPa: (**a**) transverse piezoresistor and (**b**) longitudinal piezoresistor in case of AG- Top ($\bar{d_{g}}$ = 0.8). (Only one-half of the domain displayed).

The stress difference distributions of the sensor with the annular groove AG on the top of the diaphragm (AG−Top) is investigated in Figure 16 where the distributions of $\sigma_{l}-\sigma_{t}$ at the transverse and longitudinal piezoresistors are displayed. 

**Figure 17:**
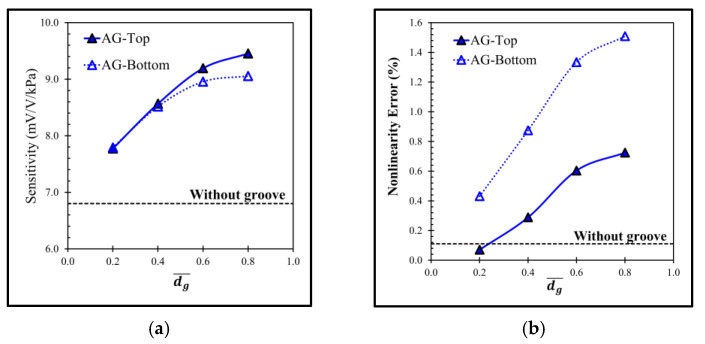
Variations of (**a**) sensitivity and (**b**) nonlinearity error with dimensionless groove depth of sensors with annular grove AG created on the top (solid line) and at the bottom (dotted line) of diaphragm.

It clearly shows that the increment of $\bar{d_{g}}$ causes the magnitude of $\sigma_{l}-\sigma_{t}$ higher leading to the higher sensitivity of the sensor as shown in Figure 17. However, the increment of $\bar{d_{g}}$ also makes the nonlinearity error higher with the faster rate compared with the sensitivity, meaning that the tradeoff between sensitivity and linearity is acute with the annular groove AG.

**Figure 18:**
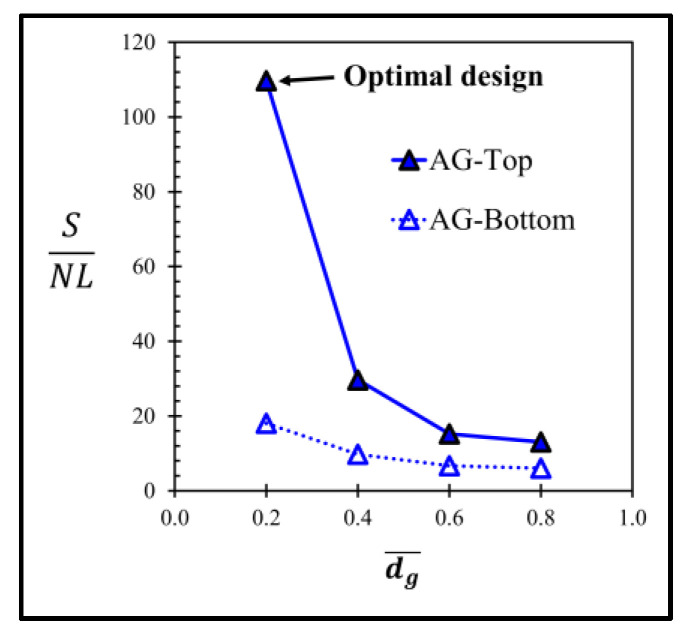
Variations of ratios of sensitivity to nonlinearity error $\left(\frac{S}{NL}\right)$ with dimensionless groove depth of sensors with annular groove AG.

The performances of the sensor with the annular groove AG at the bottom of the diaphragm are also investigated. The simulation results reveal that both sensitivity and nonlinearity error are worse compared with those on the top of the diaphragm as shown in Figure 17. Obviously, the optimal design of the annular groove AG is AG−Top at $\bar{d_{g}}$ = 0.2 as indicated by the ${\left(\frac{S}{NL}\right)}_{max}$ in Figure 18.

## 4. Groove Design Comparison

The performances of the sensors with three optimal groove designs, i.e., LG1−L0 with $\bar{d_{g}}$ = 0.2, LG2−LT−Top with $\bar{d_{g}}$ = 0.6 and $\bar{l_{g}}$ = 0.35 and AG−Top at $\bar{d_{g}}$ = 0.2, are compared in this section. According to Equation (3), the output voltage at each measurement point $\left(V_{out,i}\right)$ can be calculated by

(13) $$V_{out,i}=\left[\frac{\Delta \sigma_{1,i}-\Delta \sigma_{2,i}}{\left(4/\pi_{44}\right)+\Delta \sigma_{1,i}+\Delta \sigma_{2,i}}\right]V_{in}$$

where the subscript i denotes the values of any parameter at each measurement point of the applied pressure (P = 1, 2, 3, 4 and 5 kPa), and the input voltage ($V_{in}$) of 5 V is used in the present work. Therefore, the sensitivity can be calculated by substituting $V_{out,5}$ into $V_{max}$ in Equation (4), i.e., $S=\frac{V_{max}-V_{offset}}{\left(P_{max}-P_{0}\right)\cdot V_{in}}$, where the values of $V_{offset}$, $P_{max}$ and $P_{0}$ are 0 V, 5 kPa and 0 kPa, respectively.

**Figure 19:**
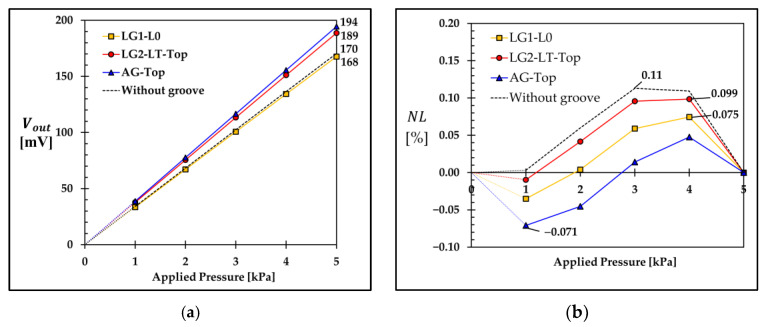
Variations of (**a**) output voltage and (**b**) nonlinearity error with the applied pressures of 1–5 kPa of sensors with three optimal groove designs (LG1−L0 with $\bar{d_{g}}$ = 0.2, LG2−LT−Top with $\bar{d_{g}}$ = 0.6 and $\bar{l_{g}}$ = 0.35 and AG−Top with $\bar{d_{g}}$ = 0.2) and without groove.

Figure 19a shows the variation of the output voltage with the applied pressures of 1–5 kPa of the sensors with and without groove. When the value of $V_{offset}$ is zero, the highest full-span scale output voltage ($V_{FSS})$ is given by the sensor with AG−Top at $V_{FSS}$ = 194 mV, where the sensor with LG2−LT−Top, the sensor without groove and the sensor with LG1−L0 give $V_{FSS}$ = 189 mV, 170 mV and 168 mV, respectively.

The nonlinearity error at each measurement point ($NL_{i}$) in Figure 19b can be calculated by

(14) $$NL_{i}=100\%\times \frac{1}{V_{FSS}}\left[V_{out,i}-V_{offset}-\left(P_{i}-P_{0}\right)\left(\frac{V_{max}-V_{offset}}{P_{max}-P_{0}}\right)\right]$$

In Figure 19b, the sensor with AG−Top gives the lowest value of the maximum nonlinearity error of 0.071%, where the sensors with LG1−L0 and LG2−LT−Top and the sensor without groove give the maximum nonlinearity error of 0.075% FSS, 0.099% FSS and 0.11% FSS, respectively. The sensitivity and nonlinearity error of the sensors with three optimal groove designs are compared with those of the sensor without groove in Table 2. The sensitivity values of the sensors with AG−Top (7.774 mV/V/kPa) and LG2−LT−Top (7.547 mV/V/kPa) are 14% and 11% higher than that of the sensor without groove, respectively, where the sensitivity of the sensor with LG1−L0 (6.707 mV/V/kPa) is 1.4% lower than that of the sensor without groove. According to the nonlinearity error comparison, the nonlinearity errors of these three sensors with AG−Top, LG1−L0 and LG2−LT−Top are 35%, 32% and 10% lower than that of the sensor without groove, respectively.

**Figure 20:**
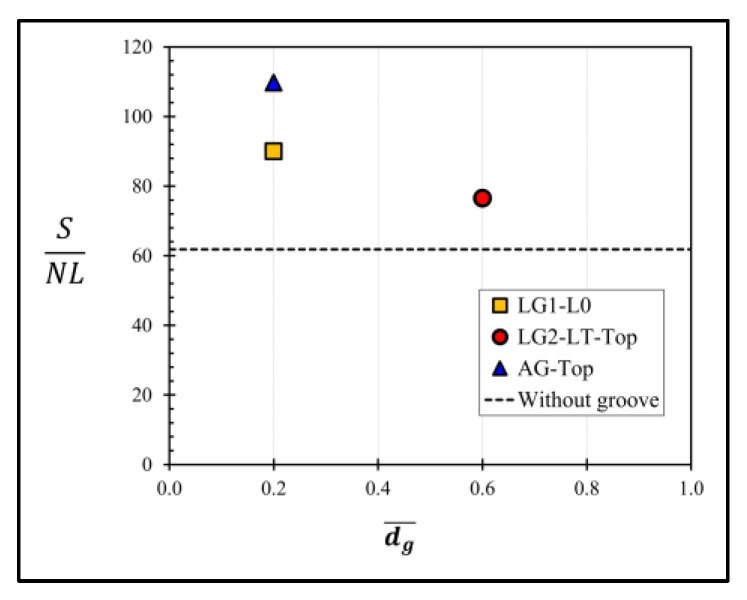
Ratios of sensitivity to nonlinearity error $\left(\frac{S}{NL}\right)$ of sensors with three optimal groove designs (LG1−L0 with $\bar{d_{g}}$ = 0.2, LG2−LT−Top with $\bar{d_{g}}$ = 0.6 and $\bar{l_{g}}$ = 0.35 and AG−Top with $\bar{d_{g}}$ = 0.2) and without a groove.

Figure 20 shows the comparison of the $\frac{S}{NL}$ of the sensors with and without groove, where the annular groove AG−Top gives the highest $\frac{S}{NL}$ of 109.49, whereas the sensor with LG1−L0, the sensor with LG2−LT−Top and the sensor without groove give those of 89.43, 76.23 and 61.82, respectively. The comparison of $\frac{S}{NL}$ indicates that all the sensors with three optimal groove designs in the present work provide higher performance than that of the sensor without groove. When LG1−L0 is created, the sensitivity is reduced because of the reduction of the averaged stress difference on the piezoresistors. Therefore, there are only two optimal groove designs, i.e., AG−Top with $\bar{d_{g}}$ = 0.2 and LG2−LT−Top with $\bar{d_{g}}$ = 0.6 and $\bar{l_{g}}$ = 0.35, that can improve both sensitivity and linearity over that of the sensor without a groove.

Table 3 summarizes the performances of MEMS piezoresistive pressure sensors in the present work and previous works. Compared with the MEMS piezoresistive pressure sensors with groove in the previous works, the MEMS piezoresistive pressure sensors with the optimal groove design of LG2−LT−Top and AG−Top in the present work have higher sensitivity and linearity.

## 5. Functional Forms of Averaged Stress Difference and Maximum Deflection of Sensor with Groove

The functional forms of the averaged stress difference of $R_{1}$ and $R_{2}$, i.e., $\Delta \sigma_{1}$ and $\Delta \sigma_{2}$, and the maximum deflection $\left(\delta_{max}\right)$ are constructed for the sensors with two groove designs that obtain higher sensitivity and lower nonlinearity error than the sensor without groove, i.e., AG−Top with $\bar{d_{g}}$ = 0.2 and LG2−LT−Top with $\bar{d_{g}}$ = 0.6 and $\bar{l_{g}}$ = 0.35. According to Equations (9)–(11), the stresses are governed by the deflection. Therefore, the functional form of the deflection of the sensor with groove in each design must be obtained first. For the sensor with the local groove LG2−LT−Top with $\bar{d_{g}}$ = 0.6 and $\bar{l_{g}}$ = 0.35, the dimensional analysis is used to determine the dimensionless form of $\delta_{max}$. With groove, $\delta_{max}$ is a function of P and $d_{g}$, but only these three variables are not able to form the dimensionless parameter. According to Equation (6), without groove, the flexural stiffness $\left(D=\frac{Ej^{3}}{12\left(1-\nu^{2}\right)}\right)$ and the diaphragm width (b) are used to construct the dimensionless deflection parameter. Therefore, these five variables, i.e., $\delta_{max}$, P, $d_{g}$, D and b, can be used in the case of having groove to form three dimensionless parameters, as follows:

(15) $$\frac{\delta_{max}}{d_{g}}=f\left(\frac{b}{d_{g}},\;\frac{12P\left(1-\nu^{2}\right)d_{g}^{3}}{Ej^{3}}\right)$$

**Figure 21:**
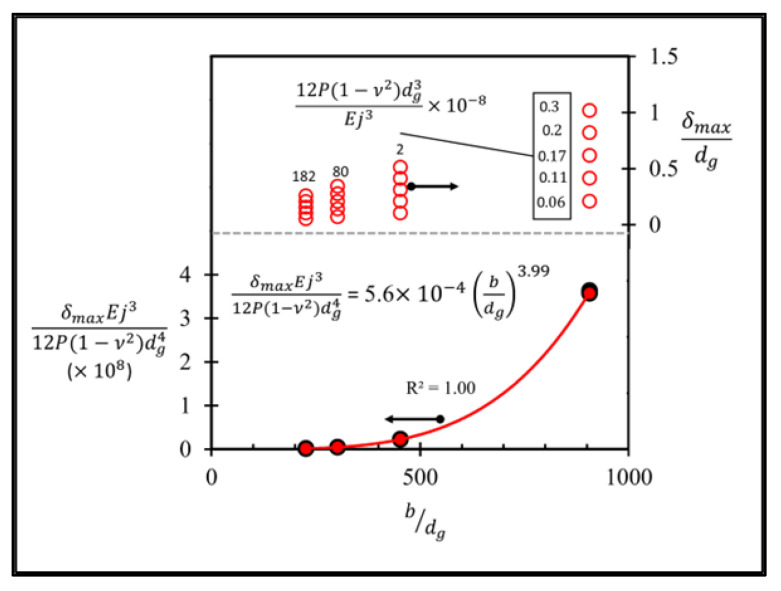
Variations of three dimensionless parameters in the case of sensor with local groove LG2−LT−Top at $\bar{l_{g}}=0.35$.

Figure 21 (upper) shows that in the variation of $\frac{\delta_{max}}{d_{g}}$ with $\frac{b}{d_{g}}$, $\frac{12P\left(1-\nu^{2}\right)d_{g}^{3}}{Ej^{3}}$ changes linearly with $\frac{\delta_{max}}{d_{g}}$ at each particular point of $\frac{b}{d_{g}}$. Therefore, $\frac{\delta_{max}}{d_{g}}$ can be divided by $\frac{12P\left(1-\nu^{2}\right)d_{g}^{3}}{Ej^{3}}$ to simply form a function involving only two dimensionless parameters, i.e., $\frac{\delta_{max}Ej^{3}}{12P\left(1-\nu^{2}\right)d_{g}^{4}}$ and $\frac{b}{d_{g}}$, and $\frac{\delta_{max}Ej^{3}}{12P\left(1-\nu^{2}\right)d_{g}^{4}}$ will represent the dimensionless maximum deflection $\left(\bar{\delta_{max}}\right)$ hereafter. The curve is then constructed to fit through a set of data on these two dimensionless parameters by using the power law, as shown in Figure 21 (lower), where the exponent and the coefficient of $\frac{b}{d_{g}}$ are 3.99 and 5.6 × 10^−4^, respectively. Therefore, the functional form of $\delta_{max}$ of the sensor with the local groove LG2−LT−Top with $\bar{l_{g}}$ = 0.35 can be expressed as

(16) $$\delta_{max,\;LG2-LT-Top}=6.72\times 10^{-3}\left(\frac{b^{3.99}d_{g}^{0.01}P\left(1-\nu^{2}\right)}{Ej^{3}}\right)$$

where the unit of length is μm and the units of the applied pressure and Young’s modulus are MPa. Equation (16) shows that the exponent of $d_{g}$ is very small because the effect of $d_{g}$ on the maximum deflection is very low in this case study. However, $d_{g}$ cannot be neglected because the groove is present and the effect of $d_{g}$ on $\delta_{max}$ is needed.

**Figure 22:**
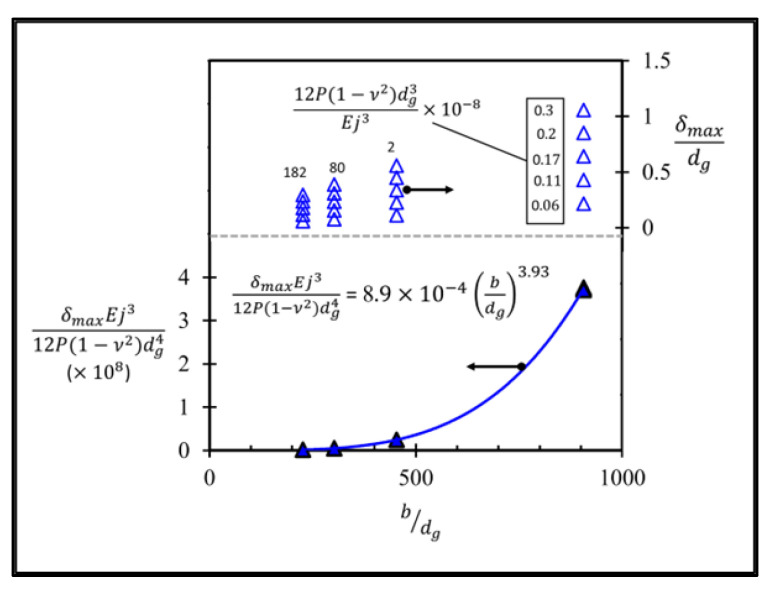
Variations of three dimensionless parameters in the case of sensor with annular groove AG−Top.

Similarly, the functional form of $\delta_{max}$ of the sensor with the annular groove AG−Top can be expressed, according to Figure 22, as

(17) $$\delta_{max,\;AG-Top}=10.71\times 10^{-3}\left(\frac{b^{3.93}d_{g}^{0.07}P\left(1-\nu^{2}\right)}{Ej^{3}}\right)$$

**Figure 23:**
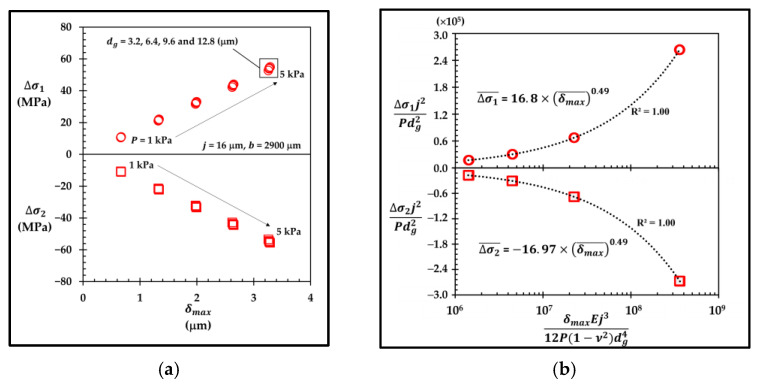
(**a**) Variations of averaged stress differences of longitudinal piezoresistor $\left(\Delta \sigma_{1}\right)$ and transverse piezoresistor $\left(\Delta \sigma_{2}\right)$ with maximum deflection ($\delta_{max}$ ) and (**b**) variations of dimensionless averaged stress differences of longitudinal piezoresistor ($\bar{\Delta \sigma_{1}}$ ) and transverse piezoresistor ($\bar{\Delta \sigma_{2}}$ ) with dimensionless maximum deflection $\left(\bar{\delta_{max}}\right)$ from two datasets (each with 20 points) in each averaged stress difference in the case of sensor with local groove LG2−LT−Top at $\bar{l_{g}}=0.35$.

**Figure 24:**
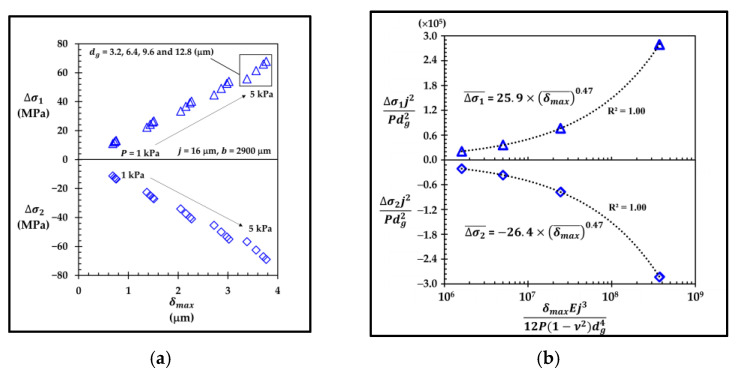
(**a**) Variations of averaged stress differences of longitudinal piezoresistor $\left(\Delta \sigma_{1}\right)$ and transverse piezoresistor $\left(\Delta \sigma_{2}\right)$ with maximum deflection ($\delta_{max}$ ) and (**b**) variations of dimensionless averaged stress differences of longitudinal piezoresistor ($\bar{\Delta \sigma_{1}}$ ) and transverse piezoresistor ($\bar{\Delta \sigma_{2}}$ ) with dimensionless maximum deflection $\left(\bar{\delta_{max}}\right)$ from two datasets (each with 20 points) in each averaged stress difference in the case of sensor with annular groove AG−Top.

Before constructing the dimensionless forms of $\Delta \sigma_{1}$ and $\Delta \sigma_{2}$ with groove, the dimensionless form of the stress without groove, in Equation (12), should be considered first. Because P and $d_{g}$ are the main input parameters when groove is involved, the ratio of the diaphragm thickness to the diaphragm width (j/b) is replaced by $1/\bar{d_{g}}$ or $j/d_{g}$ because the magnitude of Δσ increases as $d_{g}$ is increased, according to Figure 23a and Figure 24a. Therefore, the dimensionless form of Δσ can be written as

(18) $$\bar{\Delta \sigma }=\frac{\Delta \sigma }{P}\cdot {\left(\frac{j}{d_{g}}\right)}^{2}$$

To determine the variation of $\bar{\Delta \sigma_{1}}$ with $\bar{\delta_{max}}$, Figure 23a shows a dataset of twenty points of $\Delta \sigma_{1}$ versus $\delta_{max}$ in the case of the sensor with the local groove LG2−LT−Top at $\bar{l_{g}}$ = 0.35, where the data can collapse into four points when $\bar{\Delta \sigma_{1}}=\frac{\Delta \sigma_{1}}{P}\Delta \frac{j^{2}}{d_{g}^{2}}$ is plotted against $\bar{\delta_{max}}=\frac{\delta_{max}Ej^{3}}{12P\left(1-\nu^{2}\right)d_{g}^{4}}$ in Figure 23b. The power law is used to construct the curve to fit through the data in order to find the coefficient and the exponent of $\bar{\delta_{max}}$, as shown in Figure 23b, where $\bar{\Delta \sigma_{1}}$ can be expressed as a function of $\bar{\delta_{max}}$ as

(19) $${\bar{\Delta \sigma_{1}}}_{,LG2-LT-Top}=16.8\times {\left(\bar{\delta_{max}}\right)}^{0.49}$$

which can be rewritten as

(20) $${\left(\frac{\Delta \sigma_{1}}{P}\cdot \frac{j^{2}}{d_{g}^{2}}\right)}_{LG2-LT-Top}=16.8\times {\left(\frac{\delta_{max}Ej^{3}}{12P\left(1-\nu^{2}\right)d_{g}^{4}}\right)}^{0.49}$$

According to Equation (16), after substituting $\delta_{max,\;LG2-LT-Top}$ into $\delta_{max}$ in Equation (20), the functional form of $\Delta \sigma_{1}{}_{,LG2-LT-Top}$ is expressed as

(21) $$\Delta \sigma_{1}{}_{,LG2-LT-Top}=0.43\times P\left[\frac{d_{g}^{0.045}b^{1.955}}{j^{2}}\right]$$

According to Figure 23b, $\frac{\bar{\Delta \sigma_{2}}}{\bar{\Delta \sigma_{1}}}$ = −1.01 so that $\Delta \sigma_{2}{}_{,LG2-LT-Top}$ can be determined as a function of $\Delta \sigma_{1}{}_{,LG2-LT-Top}$ as

(22) $$\Delta \sigma_{2}{}_{,LG2-LT-Top}=-1.01\times \Delta \sigma_{1}{}_{,LG2-LT-Top}\;$$

The functional forms of the stress differences of the sensor with the annular groove AG−Top, i.e., $\Delta \sigma_{1}{}_{,AG-Top}$ and $\Delta \sigma_{2}{}_{,AG-Top}$, can also be found in the same way as those of $\Delta \sigma_{1}{}_{,LG2-LT-Top}$ and $\Delta \sigma_{2}{}_{,LG2-LT-Top}$. The datasets of $\Delta \sigma_{1}$ and $\Delta \sigma_{2}$ versus $\delta_{max}$ in Figure 24a are used to construct ${\bar{\Delta \sigma_{1}}}_{,AG-Top}$ and ${\bar{\Delta \sigma_{2}}}_{,AG-Top}$ versus $\bar{\delta_{max}}$ in Figure 24b.

According to Figure 24b, the functional forms of ${\bar{\Delta \sigma_{1}}}_{,AG-Top}=25.9\times {\left(\bar{\delta_{max}}\right)}^{0.49}$ and ${\bar{\Delta \sigma_{2}}}_{,AG-Top}=-26.4\times {\left(\bar{\delta_{max}}\right)}^{0.49}$ are obtained. Therefore, $\Delta \sigma_{1}{}_{,AG-Top}$ can be expressed as

(23) $$\Delta \sigma_{1}{}_{,\;AG-Top}=0.956\times P\left[\frac{d_{g}^{0.15}b^{1.85}}{j^{2}}\right]$$

whereas $\Delta \sigma_{2}{}_{,AG-Top}$ can be expressed as

(24) $$\Delta \sigma_{2}{}_{,AG-Top}=-1.02\times \Delta \sigma_{1}{}_{,AG-Top}\;$$

**Figure 25:**
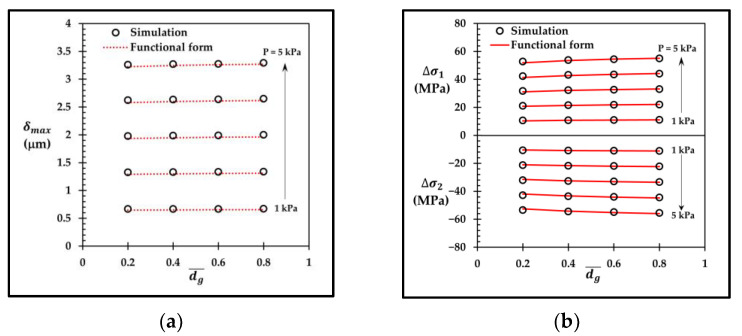
Comparisons of simulation results with (**a**) functional form of maximum deflection $\delta_{max,\;LG2-LT-Top}$ and (**b**) functional forms of averaged stress differences $\Delta \sigma_{1}{}_{,LG2-LT-Top}$ and $\Delta \sigma_{2}{}_{,LG2-LT-Top}$.

**Figure 26:**
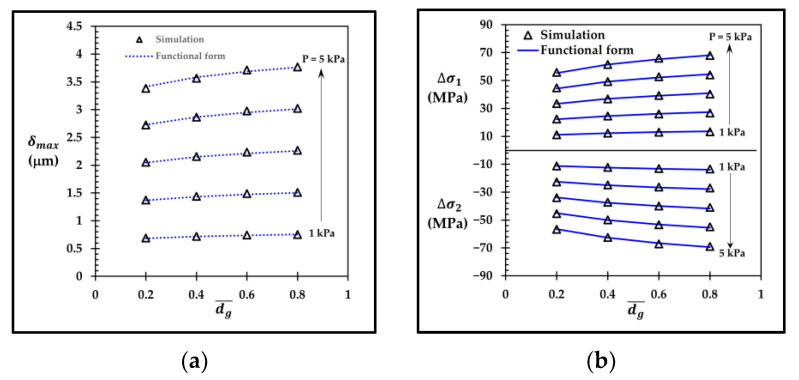
Comparisons of simulation results with (**a**) functional form of maximum deflection $\delta_{max,\;AG2-Top}$ and (**b**) functional forms of averaged stress differences $\Delta \sigma_{1}{}_{,AG-Top}$ and $\Delta \sigma_{2}{}_{,AG-Top}$.

To validate the functional forms of $\delta_{max}$, $\Delta \sigma_{1}$ and $\Delta \sigma_{2}$, the comparisons between those functional forms and the corresponding simulation results are shown in Figure 25 and Figure 26 for the sensors with the local groove LG2−LT−Top and with the annular groove AG−Top, respectively. Figure 25a,b show that $\delta_{max,\;LG2-LT-Top}$, $\Delta \sigma_{1}{}_{,LG2-LT-Top}$ and $\Delta \sigma_{2}{}_{,LG2-LT-Top}$, calculated by Equations (16), (21) and (22) respectively, are in good agreement with the simulation results, where the maximum error of $\delta_{max,\;LG2-LT-Top}$, around 2%, is found at $\bar{d_{g}}$ = 0.2 at the applied pressure of 1 kPa, and the maximum errors of $\Delta \sigma_{1}{}_{,LG2-LT-Top}$ and $\Delta \sigma_{2}{}_{,LG2-LT-Top}$, around 1.8%, are found at $\bar{d_{g}}$ = 0.2 at the applied pressure of 3 kPa. Figure 26a,b show that $\delta_{max,\;AG-Top}$, $\Delta \sigma_{1}{}_{AG-Top}$ and $\Delta \sigma_{2}{}_{AG-Top}$, calculated by Equations (17), (23) and (24) respectively, are also in good agreement with the simulation results, where the maximum error of $\delta_{max,\;AG-Top}$, around 1.8%, is found at $\bar{d_{g}}$ = 0.6 at the applied pressure of 3 kPa, and the maximum errors of $\Delta \sigma_{1}{}_{,AG-Top}$ and $\Delta \sigma_{2}{}_{,AG-Top}$, around 1%, are found at $\bar{d_{g}}$ = 0.6 at the applied pressure of 5 kPa.

The functional forms of the averaged stress differences in terms of $d_{g}$ in Equations (21)–(24), i.e., $\Delta \sigma_{1}{}_{,LG2-LT-Top}$, $\Delta \sigma_{2}{}_{,LG2-LT-Top}$, $\Delta \sigma_{1}{}_{,\;AG-Top}$ and $\Delta \sigma_{2}{}_{,AG-Top}$, respectively, can be used to calculate $V_{out,i}$ by substituting $\Delta \sigma_{1}$ and $\Delta \sigma_{2}$ in the case study of interest into Equation (13). Therefore, sensitivity can be calculated by substituting $V_{out,max}$ into Equation (4) while nonlinearity error can be calculated by substituting $V_{out,i}$ and $V_{out,max}$ into Equation (14).

## 6. Discussion

**Figure 27:**
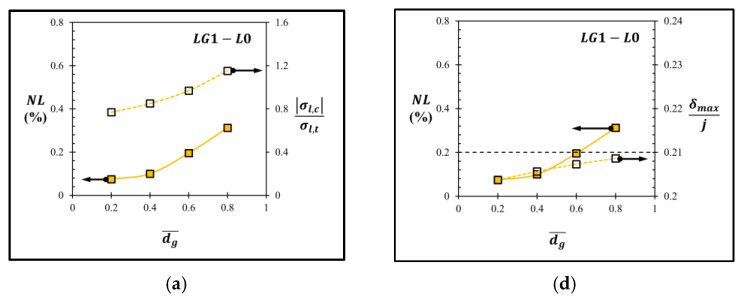

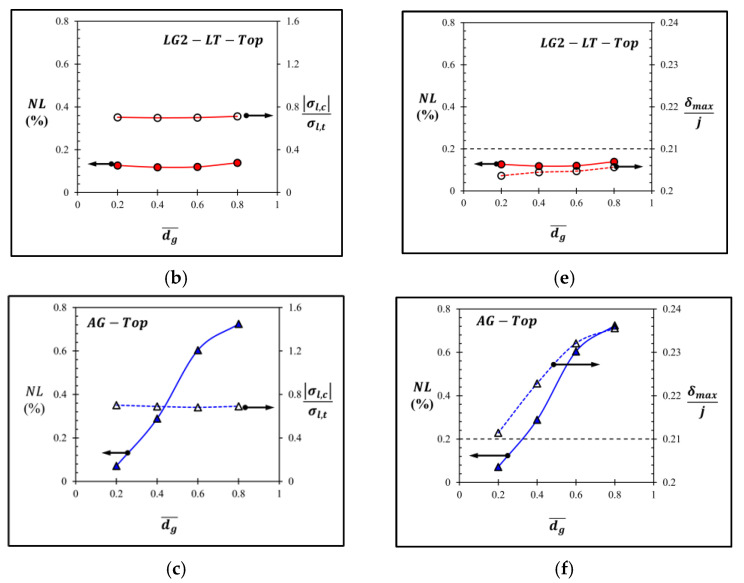
Variations of nonlinearity error with the ratio of the compression stress to the tension stress $\left(\frac{\left|\sigma_{l,c}\right|}{\sigma l,t}\right)$ versus $\bar{d_{g}}$ in the longitudinal stress direction in the case of (**a**) LG1−L0, (**b**) LG2−LT−Top at $\bar{l_{g}}=0.35$ and (**c**) AG−Top. Variations of nonlinearity error and the ratio of maximum deflection to diaphragm thickness $\left(\delta_{max}/j\right)$ versus $\bar{d_{g}}$ in the case of (**d**) LG1−L0, (**e**) LG2−LT−Top at $\bar{l_{g}}=0.35$, and (**f**) AG−Top.

Three selected groove designs, i.e., LG1−L0 with $\bar{d_{g}}$ = 0.2, LG2−LT−Top with $\bar{d_{g}}$ = 0.6 and $\bar{l_{g}}$ = 0.35 and AG−Top at $\bar{d_{g}}$ = 0.2, are investigated here. For the sensor with the local groove LG1, creating groove at the piezoresistor reduces sensitivity because the piezoresistor is moved closer to the neutral axis. Moreover, there are some concerns about fabrication techniques, such as (1) the metallization process has a high chance of failure due to the vertical deposition with high step size and (2) the geometric error due to the etching process that deteriorates the performance of the sensor. Therefore, the local groove LG1 in the present work is not preferred for use in the MEMS piezoresistive pressure sensor. However, the remaining area of the beam, where the local groove LG1 is not created, gains higher stress, as shown in Figure 7. Therefore, moving the piezoresistor to that area is preferable in order to increase the sensitivity of the sensor, but the alignment errors between the piezoresistor and the beam layer due to the fabrication process should be carefully treated, as reported by Huang and Zhang (2014) [28]. For the sensor with the local groove LG2, the stress at the piezoresistor can be made higher by creating a groove along the diaphragm edge, according to the simulation result of the annular groove AG. For the sensor with the annular groove AG, the tradeoff between sensitivity and nonlinearity error is the challenge that should be achieved. The main source of the nonlinearity error of each groove design can be investigated in Figure 27. The ratio of the compression stress to the tension stress $\left(\frac{\left|\sigma_{l,c}\right|}{\sigma l,t}\right)$ in the longitudinal stress direction, which occur in SCR on the top of the beam and at the bottom of the diaphragm, respectively, can represent the stretching effect on the piezoresistor, which is the main cause of linearity reduction. In the case of the sensors with the local grooves LG1−L0 and LG2−LT−Top, the stretching effect on the piezoresistor is the main source of linearity reduction when the ratio of the maximum deflection to the diaphragm thickness $\left(\delta_{max}/j\right)$ is lower than 0.21, which can be observed from the correlation between the nonlinearity error and $\frac{\left|\sigma_{l,c}\right|}{\sigma l,t}$ in Figure 27a,b. When $\delta_{max}/j>$ 0.21, the nonlinearity error is dominated by the balloon effect, which can be observed from the correlation between the nonlinearity error and $\delta_{max}/j\;$ in Figure 27f in the case of the sensor with the annular groove AG−Top. Therefore, the further improvement of linearity for the sensor with the annular groove AG−Top can be achieved by using the local stiffness concept to reduce $\delta_{max}$ at the center of the diaphragm to avoid the balloon effect. In the future work, dielectric layers and thermal stress will also be investigated as major concerns about high residual mechanical stresses that arise when the complex diaphragm geometry is created.

## 7. Conclusions

Three groove designs for the MEMS piezoresistive pressure sensor were investigated in the present work. There were two designs for the local groove type (LG1 and LG2) and one design for the annular groove type (AG). Three configurations of the groove location were investigated in LG1 and LG2, i.e., L0, 0T and LT. The effects of the groove location on the top and at the bottom of the diaphragm were investigated in LG2 and AG. The effects of the dimensionless groove depth variation, i.e., $\bar{d_{g}}$ = 0.2, 0.4, 0.6 and 0.8, on sensitivity and nonlinearity error were investigated in all groove designs. Because of the limitation of LG1 and AG, the effects of the dimensionless groove length variation, i.e., $\bar{l_{g}}$ = 0.175 and 0.35, on sensitivity and nonlinearity error were investigated in LG2 only, while the values of $\bar{l_{g}}$ of LG1 and AG were fixed as 0.35 and 8.0, respectively. In this study, the effect of the groove width was not included, so that the dimensionless groove width ($\bar{w_{g}}$) was fixed as 1.0 in all case studies. The finite element method was used to determine the stress and deflection of the sensor with groove, which were later used for calculating sensitivity and nonlinearity error. The simulation results revealed that the best configurations of the groove locations for LG1 and LG2 were L0 and LT, respectively, because their $\frac{S}{NL}$ values were higher than those of other groove locations in the same configuration. To obtain higher sensitivity and lower nonlinearity error, the groove must be created at the top of the diaphragm, which was the compression side, in this work. The increment of $\bar{d_{g}}$ can improve sensitivity in the case of LG2 and AG because of the higher stress difference at the piezoresistor, while sensitivity decreased in case of LG1 because the piezoresistor was moved closer to the neutral axis, leading to a lower stress difference at the piezoresistor. For the effect of $\bar{d_{g}}$ on nonlinearity error, the lowest nonlinearity error of the sensor with each groove design was found at the particular value of $\bar{d_{g}}$, i.e., $\bar{d_{g}}$ = 0.2, 0.6 and 0.2 for LG1, LG2 and AG, respectively. For the effect of $\bar{l_{g}}$ on LG2, the double increment of $\bar{l_{g}}$ (from 0.175 to 0.35) slightly improved both sensitivity and linearity. Therefore, the three optimal groove designs in the present work were LG1−L0, LG2−LT−Top and AG−Top with ($\bar{d_{g}}$, $\bar{l_{g}}$) = (0.2, 0.35), (0.6, 0.35) and (0.2, 8.0), respectively. In the case of the optimal groove design, the sensitivity values of the sensors with LG1−L0, LG2−LT−Top and AG−Top were 6.707 mV/V/kPa, 7.547 mV/V/kPa and 7.774 mV/V/kPa, respectively, while the nonlinearity errors of those sensors were 0.075% FSS, 0.099% FSS and 0.071% FSS, respectively. From the comparisons of the sensitivity and nonlinearity error of the sensors with and without groove, there were only two optimal groove designs that helped to improve both sensitivity and linearity, i.e., LG2−LT−Top and AG−Top. The sensitivity of the sensors with LG2−LT−Top and AG−Top were 11% and 14% higher than that of the sensor without groove, respectively, while the nonlinearity errors of those sensors were 10% and 35% lower than that of the sensor without groove, respectively. Although the sensor performances of AG−Top were slightly better than those of LG2−LT−Top, the net groove volume of AG−Top ($\bar{d_{g}}\cdot \bar{l_{g}}\cdot \bar{w_{g}}\times 4=6.4$) was around four times greater than that of LG2−LT−Top ($\bar{d_{g}}\cdot \bar{l_{g}}\cdot \bar{w_{g}}\times 8=1.68$), and hence, more effort in fabrication was required. Finally, the functional forms of the averaged stress differences of the longitudinal ($\Delta \sigma_{1}$) and transverse ($\Delta \sigma_{2}$) piezoresistors and the maximum deflection of the diaphragm ($\delta_{max}$) of the sensors with the optimal groove designs of LG2−LT−Top and AG−Top were constructed. By accounting for the exponent values of the groove depth ($d_{g}$) in the functional forms of $\Delta \sigma_{1}$ and $\delta_{max}$, it was found that $d_{g}$ increased the averaged stress difference at the faster rate than $\delta_{max}$ in both sensors with LG2−LT−Top and AG−Top.

## Figures and Tables

**Table 1:** List of groove configurations investigated in the present work.

Configuration	Groove Depth $\left(\bar{d_{g}}\right)$	Groove Length $\left(\bar{l_{g}}\right)$
LG1−L0	0.2, 0.4, 0.6 and 0.8	0.35
LG1−0T	0.2, 0.4, 0.6 and 0.8	0.35
LG1−LT	0.2, 0.4, 0.6 and 0.8	0.35
LG2−L0−Top	0.2, 0.4, 0.6 and 0.8	0.175 and 0.35
LG2−0T−Top	0.2, 0.4, 0.6 and 0.8	0.175 and 0.35
LG2−LT−Top	0.2, 0.4, 0.6 and 0.8	0.175 and 0.35
LG2−L0−Bottom	0.2, 0.4, 0.6 and 0.8	0.175 and 0.35
LG2−0T−Bottom	0.2, 0.4, 0.6 and 0.8	0.175 and 0.35
LG2−LT−Bottom	0.2, 0.4, 0.6 and 0.8	0.175 and 0.35
AG−Top	0.2, 0.4, 0.6 and 0.8	8.0
AG−Bottom	0.2, 0.4, 0.6 and 0.8	8.0

**Table 2:** Comparisons between sensitivity and nonlinearity of the sensors with three optimal groove designs and those of the sensor without groove.

Groove Design	S [mV/V/kPa]	$NL{\;}_{max}$ [% FSS]
LG1−L0 $\mathrm{with}\;\bar{d_{g}}$ = 0.2	6.707	0.075
	Decrease (−1.4%)	Decrease (−32%)
LG2−LT−Top $\mathrm{with}\;\bar{d_{g}}$$=0.6\;\mathrm{and}\;\bar{l_{g}}$ = 0.35	7.547	0.099
	Increase (11%)	Decrease (−10%)
AG−Top $\mathrm{with}\;\bar{d_{g}}$ = 0.2	7.774	0.071
	Increase (14%)	Decrease (−35%)
Without a groove, according to Thawornsathit et al., 2022 [41]	6.8	0.11

**Table 3:** Comparison of performance parameters of different MEMS piezoresistive pressure sensors.

MEMS Piezoresistive Pressure Sensor	Pressure Range (kPa)	Diaphragm Width	S (mV/V/kPa)	NL (% FSS)	S/NL
Tran et al. (2018b) [20] (Local groove)	0–5	2900 μm	6.93	0.23	30.15
Li et al. (2020) [21] (Annular groove)	0–6.895	3600 μm	4.48	0.25	17.92
Sahay et al. (2021) [38] (Annular groove)	0–5	3600 μm	4.061	0.15	27.07
Present work, LG1−L0$\;(\bar{l_{g}}=0.35$$\;\mathrm{and}\;\bar{d_{g}}=0.2$)	1–5	2900 μm	6.707	0.075	89.43
Present work, LG2−LT−Top$\;(\bar{l_{g}}=0.35$$\;\mathrm{and}\;\bar{d_{g}}=0.6$)	1–5	2900 μm	7.547	0.099	76.23
Present work, AG−Top$\;(\bar{l_{g}}=8$$\;\mathrm{and}\;\bar{d_{g}}=0.2$)	1–5	2900 μm	7.774	0.071	109.49

## Data Availability

Not applicable.
